# BG-4, a novel anticancer peptide from bitter gourd (*Momordica charantia*), promotes apoptosis in human colon cancer cells

**DOI:** 10.1038/srep33532

**Published:** 2016-09-15

**Authors:** Vermont P. Dia, Hari B. Krishnan

**Affiliations:** 1 Department of Food Science and Technology, The University of Tennessee Institute of Agriculture, Knoxville TN 37996 USA; 2 USDA-ARS Plant Genetics Resources Unit, University of Missouri, Columbia, MO 65211 USA

## Abstract

Momordica charantia is a perennial plant with reported health benefits. BG-4, a novel peptide from *Momordica charantia,* was isolated, purified and characterized. The trypsin inhibitory activity of BG-4 is 8.6 times higher than purified soybean trypsin inhibitor. The high trypsin inhibitory activity of BG-4 may be responsible for its capability to cause cytotoxicity to HCT-116 and HT-29 human colon cancer cells with ED_50_ values of 134.4 and 217.0 μg/mL after 48 h of treatment, respectively. The mechanism involved in the cytotoxic effect may be associated with induction of apoptosis as evidenced by increased percentage of HCT-116 and HT-29 colon cancer cells undergoing apoptosis from 5.4% (untreated) to 24.8% (BG-4 treated, 125 μg/mL for 16 h) and 8.5% (untreated) to 31.9% (BG-4 treated, 125 μg/mL for 16 h), respectively. The molecular mechanistic explanation in the apoptosis inducing property of BG-4 is due to reduced expression of Bcl-2 and increased expression of Bax leading to increased expression of caspase-3 and affecting the expression of cell cycle proteins p21 and CDK2. This is the first report on the anti-cancer potential of a novel bioactive peptide isolated from *Momordica charantia in vitro* supporting the potential therapeutic property of BG-4 against colon cancer that must be addressed using *in vivo* models of colon carcinogenesis.

Colorectal cancer (CRC) is the third most common cancer in the world accounting for 1.36 million cases and 694,000 deaths in 2012 according to the most recent GLOBOCAN report^1^. Sporadic CRC which accounts for a majority of cases involve genetic mutations leading to conversion of epithelial cells to adenocarcinoma and carcinoma^2^. The stage of the disease at the time of diagnosis largely determines prognosis with 5-year survival rate of 90.3, 70.4 and 12.5% for patients diagnosed with localized, regional and distant tumors^3^. Hence early detection through screening can largely increase survival and reduce mortality from this malignancy. One of events happening in early stage of colon tumorigenesis is the alterations in the proliferative pattern and impairment in apoptosis in the epithelial cells of colon crypts^4^. Avoidance of apoptosis (also known as programmed cell death) is one of the hallmarks of cancer^5^ and is controlled by a variety of protein machineries including proteins involved in the intrinsic mitochondrial pathway and extrinsic death receptor-mediated pathway. Regardless of the pathway involved, apoptosis is implemented by a group of cysteine-dependent aspartyl-specific protease known as caspases^6^ which are grouped into initiatior caspases including caspases 8 and 9 and executioner caspases including caspases 3, 6 and 7 7. Activation of apoptosis pathways is one of the key approaches in combatting cancer including CRC. CRC development follows a distinct sequential transformation hence has been associated with several epidemiological risk factors including age, family history and inflammatory bowel disease^2^. In addition, preventable risk factors such as obesity^8^, Western diet^9^,^10^ and sedentary lifestyle^11^ has been associated with increased risk of CRC while diet high in dietary fiber has been reported to have a protective effect against CRC^12^,^13^.

*Momordica charantia*, commonly known as bitter gourd (BG) or bitter melon, belongs to the family of Cucurbitaceae and is cultivated throughout the world, traditionally been used in folk medicines in cases of malaria, peptic ulcers, kidney stone, eczema^14^ to name a few and is commonly used in the treatment of type II diabetes^15^,^16^. In addition, extracts from different parts of BG possessed anticancer properties. MAP30, momordica anti-HIV protein, inhibited proliferation of MDA-MB-231 breast cancer cells and increased survival in MDA-MB-231 breast cancer mice xenografts^17^, methanolic extract of BG led to reduced proliferation and activated autophagy in CRC^18^ and sensitized CRC to the cytotoxic effect of doxorubicin^19^ while BG lectin has been shown to induce G2/M cell cycle arrest, autophagy and apoptosis in hepatocellular cancer^20^. These previous studies support the idea on the potential therapeutic use of BG and its biologically active constituents.

In the present study, we have devised a simple and effective procedure to isolate low-molecular weight protease inhibitor (BG-4) from BG and demonstrate its potential therapeutic property against colon cancer cells. The objectives were to characterize this novel anticancer peptide and to assess its capability to affect the growth of two human colon cancer cell lines, HCT-116 and HT-29. We first report here the discovery of a novel anticancer peptide from *Momordica charantia* by utilizing human colon cancer cells. We showed that BG-4 is very potent in inhibiting proliferation of both colon cancer cells, promoted apoptosis as measured by flow cytometry and microscopy study and the mechanism of action involved is through downregulation of antiapoptotic proteins Bcl-2 and XIAP, upregulation of proapoptotic proteins Bax and caspase-3 and modification of cell cycle proteins p21 and cyclin dependent kinase 2 (CDK2).

## Results

### SDS-PAGE analysis of bitter gourd seed proteins

**Figure 1:**
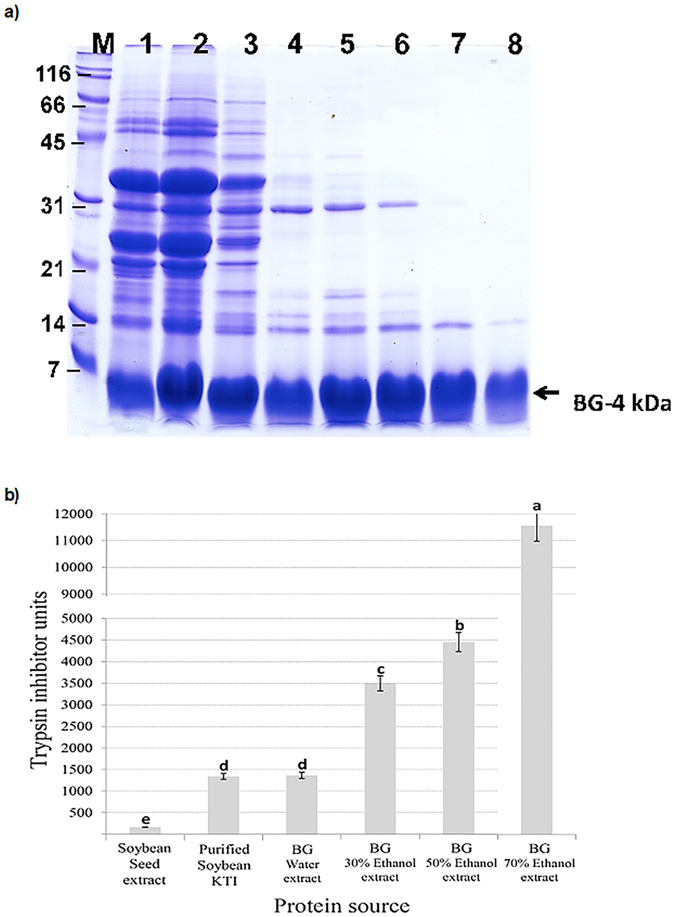
Electrophoresis profile and trypsin inhibitory activity of aqueous and ethanolic extracts from *Momordica charantia.* (**a**) SDS-PAGE profile of different *Momordica charantia* extracts indicates that increasing ethanol concentrations in the extraction medium led to isolation and purification of a novel peptide termed BG-4. (**b**) Trypsin inhibitory activity of *Momordica charantia* extracts is associated with the degree of BG-4 purity. Increasing BG-4 purity led to increased capability of the extract to inhibit trypsin, the purified BG-4 peptide from 70% ethanol extraction is 8.6 times more effective in inhibiting trypsin as compared to purified soybean Kunitz trypsin inhibitor. Mean values represented as bars with different letter(s) are significantly different from each other (P < 0.05, n > 3).

Protein profile of BG seed extracted with an increasing concentration of ethanol is shown in Fig. 1a Several polypeptides ranging from 70 to 4 kDa was seen in 0 to 20% ethanol seed extracts. Prominent among them were a 35 kDa, 24 kDa and 4 kDa proteins. These three abundant proteins accounted for the bulk of the BG seed proteins. Increasing the concentration of ethanol drastically reduced the abundance of the 35 kDa and 24 kDa proteins. Interestingly, the 4 kDa peptide was extractable with all the tested concentration of ethanol. In 60 and 70% ethanol extracts only the 4 kDa and a 14 kDa peptides were present. Our observation suggests that aqueous ethanol could be exploited as a fast and easy way of purification of the 4 kDa peptide.

### Identification of the 4 kDa protein as trypsin inhibitor

Previous studies have established the presence of low molecular weight protease inhibitors in BG seeds. Two novel trypsin inhibitors, MCTI-II’ and BGIT, have been purified from BG and their amino acid sequence have been elucidated^21^. MCTI-II’ consists of 27 amino acid residues while the BGIT is made up of 68 amino acid residues and is capable of inhibiting not only trypsin but also subtilisin Carlsberg. We measured the trypsin inhibitor activity of the aqueous ethanol extracts to verify if they exhibit any protease inhibitor activity (Fig. 1b). The aqueous ethanol extracts, which predominantly contain the 4 kDa peptide, revealed relatively high levels of trypsin inhibitor activity when compared to that of soybean seed (Fig. 1b). Additionally, MALDI-TOF-MS analysis of the 4 kDa protein followed by peptide mass searches indicated that significant peptide matches with *Momordica charantia* protease inhibitor (Table 1). These observations indicate that the abundant 4 kDa peptide, which is preferentially soluble in aqueous ethanol, is likely a trypsin inhibitor.

### BG-4 inhibited proliferation of HCT-116 and HT-29 colon cancer cells

**Figure 2:**
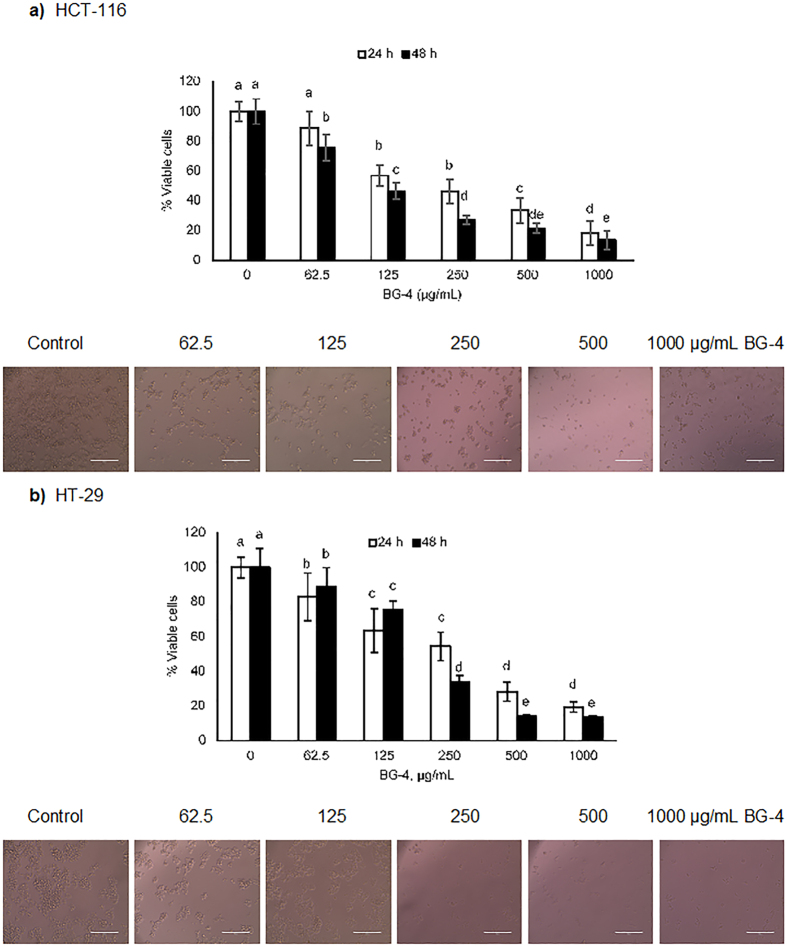
BG-4 purified from *Momordica charantia* caused a dose-dependent cytotoxicity to HCT-116 and HT-29 human colon cancer cells. (**a**) BG-4 calculated ED_50_ values for 24 and 48 h treatments were 251.3 and 134.4 μg/mL for HCT-116, respectively and (**b**) BG-4 calculated ED_50_ values for 24 and 48 h treatments were 249.2 and 217.0 μg/mL for HT-29, respectively. Representative phase-contrast images of human colon cancer cells as affected by BG-4 treatment after 48 h showed changes in the morphology of human colon cancer cells consistent with its cytotoxic activity. Scale bar is 200 μm. Mean values within cell line represented as bars with different letter(s) are statistically different from each other (P < 0.05, n > 3).

As shown in Fig. 2, BG-4 dose dependently inhibited the proliferation of HCT-116 and HT-29 colon cancer cells *in vitro*. At 250 μg/mL, HCT-116 proliferation was significantly inhibited by 53.6 and 72.8% while HT-29 proliferation was significantly inhibited by 45.6 and 65.8% after 24 and 48 h treatment, respectively. The calculated ED_50_ values for 24 and 48 h treatments were 251.3 and 134.4 μg/mL for HCT-116, respectively and 249.2 and 217.0 μg/mL for HT-29, respectively. Figure 2 also presents images of HCT-116 and HT-29 colon cancer cells as affected by BG-4 treatment for 48 h. Treatment with BG-4 led to changes in the morphology of the cells associated with the process of apoptosis such as cell shrinkage and blebbing and the cytotoxic effect of BG-4 is apparent starting at treatment concentration of 250 μg/mL.

### BG-4 inhibited colony formation of HCT-116 and HT-29 colon cancer cells

**Figure 3:**
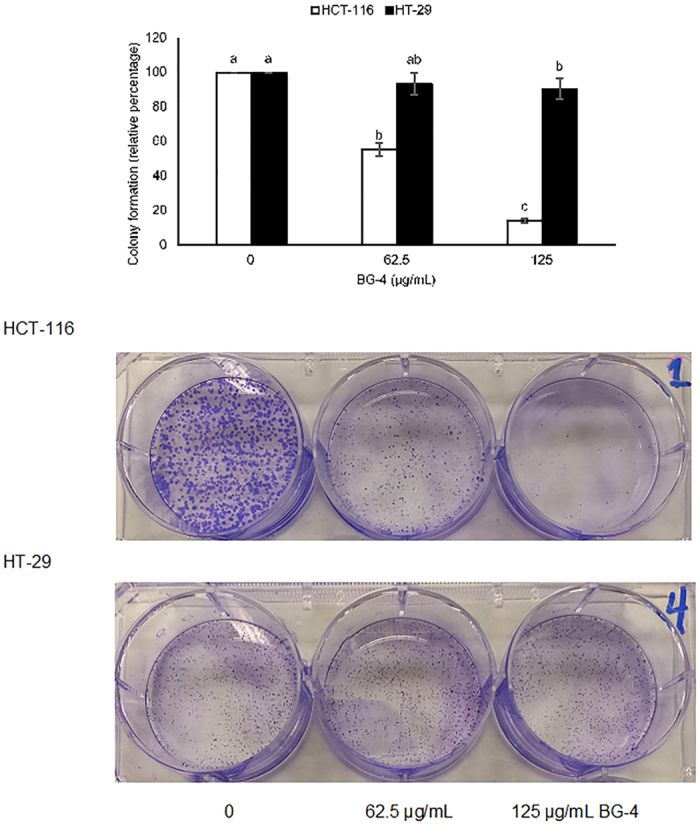
BG-4 inhibited the capability of HCT-116 and HT-29 colon cancer cells to form colony. At 125 μg/mL, BG-4 statitistically inhibited colony formation of HCT-116 and HT-29 human colon cancer cells by 85.9 and 9.4%, respectively. Representative images are shown from respective human colon cancer cells and treatment. Mean values within cell line represented as bars with different letter(s) are statistically different from each other (P < 0.05, n = 4).

Figure 3 shows the effect of BG-4 treatment on the capability of colon cancer cells to form colonies. BG-4 treatment at 62.5 and 125 μg/mL resulted in 44.6 and 85.9% significant reduction in colony formation of HCT-116 cells, respectively. On the other hand, HT-29 colony formation was only significantly reduced by 9.4% at 125 μg/mL BG-4 treatment.

### BG-4 promoted apoptosis in HCT-116 and HT-29 colon cancer cells

**Figure 4:**
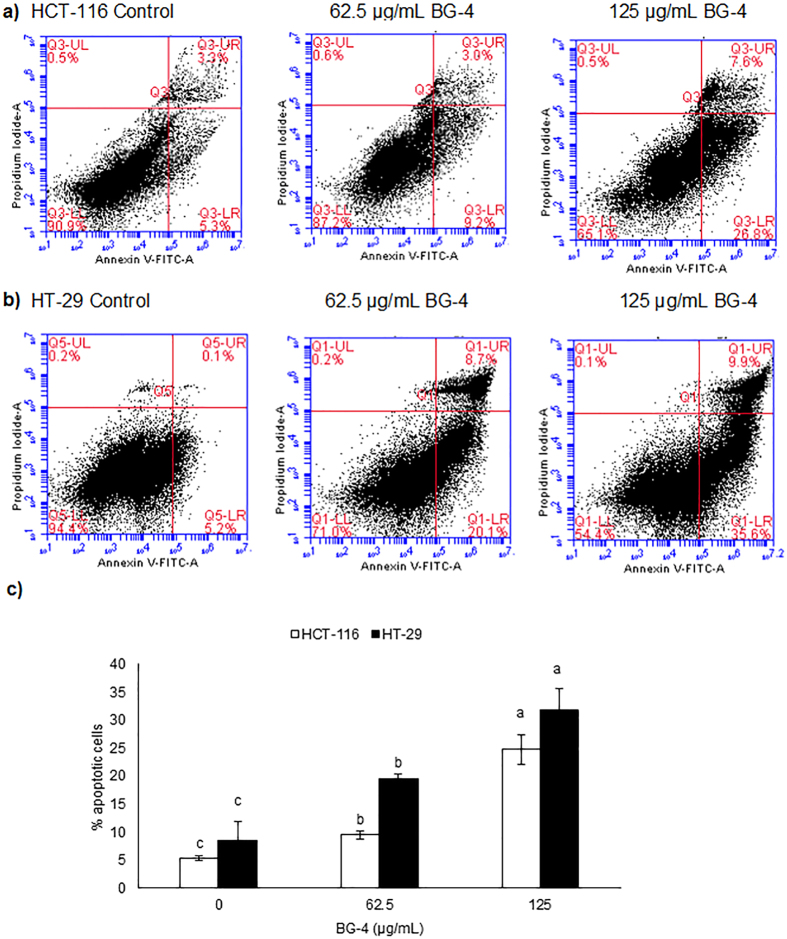
BG-4 induces apoptosis in HCT-116 and HT-29 colon cancer cells as measured by flow cytometry. (**a**) BG-4 treatment led to increase in the percentage of HCT-116 human colon cancer cells undergoing apoptosis (**b**) BG-4 treatment led to increase in the percentage of HT-29 human colon cancer cells undergoing apoptosis (**c**) Quantification of human colon cancer cells undergoing apoptosis as affected by BG-4 treatment. In HCT-116 cells, the apoptotic cells were from 5.4% (untreated) to 9.5% and 24.8% for 62.5 μg/mL and 125 μg/mL treatment, respectively while in HT-29 human colon cancer cells, the percentage of cells undergoing apoptosis was increased from 8.5% (untreated) to 19.6% and 31.9% for 62.5 μg/mL and 125 μg/mL treatment, respectively. Mean values within cell line represented as bars with different letter(s) are statistically different from each other (P < 0.05, n = 3).

**Figure 5:**
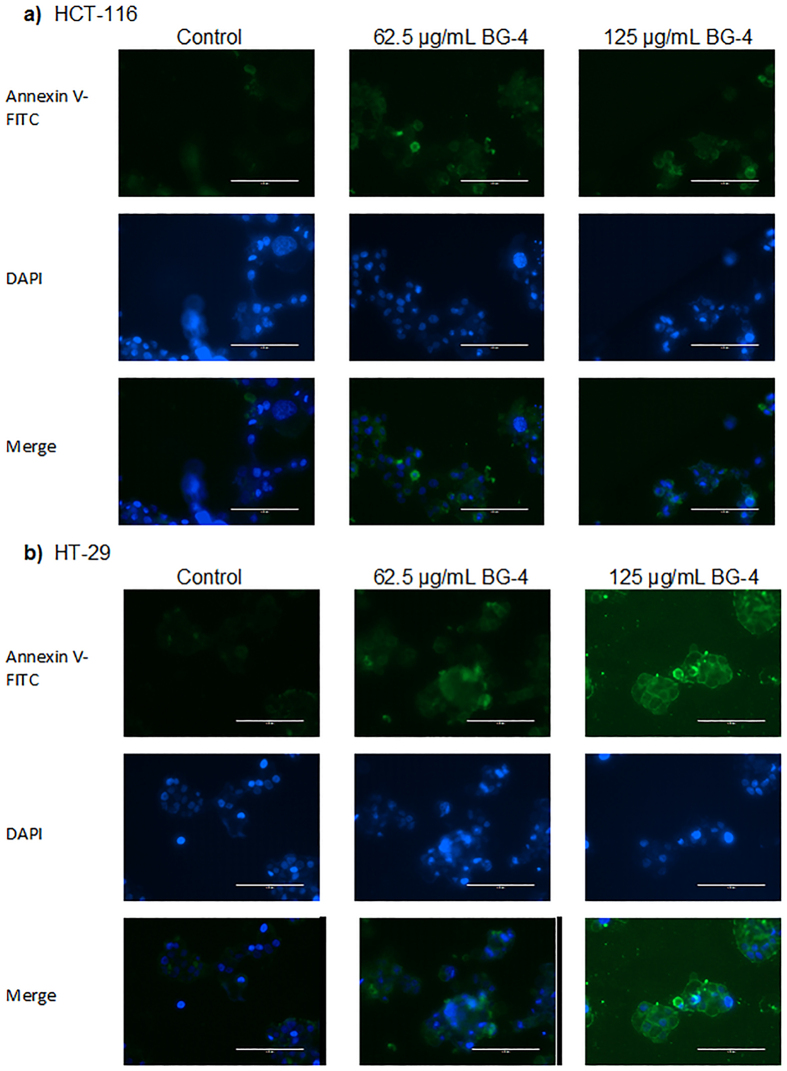
Flourescence microcopy analysis of Annexin V binding to human colon cancer cells showed that BG-4 treatment promoted apoptosis in HCT-116 and HT-29 colon cancer cells. (**a**) Representative images from at least 3 independent experiments with 6 random fields per experiment taken from untreated and BG-4 treated HCT-116 human colon cancer cells. (**b**) Representative images from at least 3 independent experiments with 6 random fields per experiment taken from untreated and BG-4 treated HT-29 human colon cancer cells. Increased green fluorescence in BG-4 treated cells indicates that cells mitochondria became permeable to Annexin V, a characteristics of cells undergoing apoptosis.

We then treated HCT-116 and HT-29 colon cancer cells with BG-4 concentrations (62.5 and 125 μg/mL) less than the calculated ED_50_ values for the corresponding cell lines for 16 h. This time point was chosen as we would like to detect cells undergoing early apoptosis and based on proliferation studies, 24 and 48 h treatments with BG-4 led to significant cytotoxic effect in the cells. As shown in Fig. 4, the concentrations of cells undergoing apoptosis (quadrant 2 in the flow cytometry figures), were dose dependently increased by BG-4 treatment. In HCT-116 human colon cancer cells, the percentage of cells undergoing apoptosis was increased from 5.4% (untreated) to 9.5% and 24.8% for 62.5 μg/mL and 125 μg/mL treatment, respectively. In HT-29 human colon cancer cells, the percentage of cells undergoing apoptosis was increased from 8.5% (untreated) to 19.6% and 31.9% for 62.5 μg/mL and 125 μg/mL treatment, respectively. This was further supported by fluorescence microscopy of cells stained with Annexin V-FITC and DAPI as shown in Fig. 5. Fluorescence microscopy images showed that BG-4 treated human colon cancer cells have higher Annexin V staining as compared with untreated human colon cancer cells supporting the data gathered from flow cytometry experiments.

### BG-4 modified the expressions of proteins associated with apoptosis and cell cycle in human colon cancer cells

**Figure 6:**
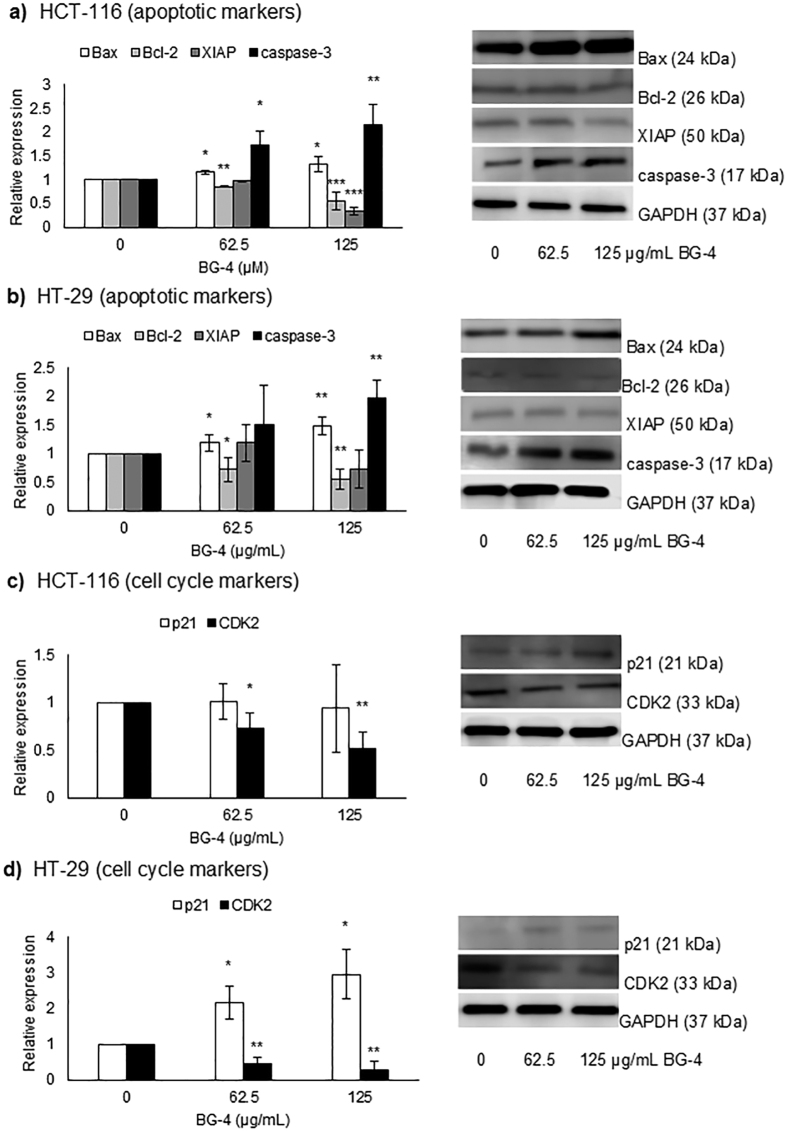
BG-4 mechanism of action in inducing apoptosis in human colon cancer cells. (**a**) In HCT-116, BG-4 promoted apoptosis by increasing Bax:Bcl-2 ratio leading to reduced expression of XIAP and increased expression of caspase-3. (**b**) In HT-29, BG-4 promoted apoptosis by increasing Bax:Bcl-2 ratio leading to increased expression of caspase-3 without significantly affecting XIAP expression. (**c**) In HCT-116, BG-4 significantly reduced the expression of CDK2 without affecting the expression of p21. (**d**) In HT-29, BG-4 increased the expression of p21 leading to reduced expression of CDK2. All analyses were done in at least three independent replicates and significance are indicated by *(P < 0.05), **(P < 0.01) and ***(P < 0.0001).

We then measured the expression of proteins associated with apoptosis in HCT-116 and HT-29 colon cancer cells. As shown in Fig. 6a, BG-4 at 125 μg/mL significantly modified the expression of different apoptotic markers in HCT-116 colon cancer cells by increasing the expression of Bax and caspase-3 by 1.3-fold and 2.2-fold, respectively and decreasing the protein expression of Bcl-2 and XIAP by 44.3% and 67.0%, respectively. Figure 6b indicates that BG-4 treatment at 125 μg/mL led to a significant increase in the expression of pro-apoptotic Bax in HT-29 colon cancer cells by 1.5-fold with concomitant reduction in the expression of anti-apoptotic protein Bcl-2 by 1.8-fold. This led to increase expression of apoptosis executioner caspase-3 by 2.0-fold without affecting the expression of anti-apoptotic protein XIAP. To further understand the anti-proliferative effect of BG-4 in human colon cancer cells, expression of protein markers associated with cell survival with particular emphasis on cell cycle proteins was measured. As shown in Fig. 6c, BG-4 at 125 μg/mL did not affect the expression of cyclin dependent kinase inhibitor p21 but reduce the expression of CDK2 by 1.9-fold in HCT-116 colon cancer cells while in HT-29 as presented in Fig. 6d, BG-4 treatment at 125 μg/mL increase the expression of p21 by 3.0-fold and decrease expression of CDK2 by 3.5-fold.

## Discussion

We report for the first time the biological activity of a newly isolated and identified bioactive peptide from *Momordica charantia* we called BG-4. BG-4 was easily extracted using different concentrations of ethanol and can be purified from the rest of BG seed proteins by 70% ethanol as evidenced by electrophoresis profile of different BG seed aqueous ethanol extracts (Fig. 1a), a method that can be easily performed for the potential application of BG-4 as an anticancer agent. Amino acid sequencing of the purified peptide indicated that BG-4 amino acid sequences matched with the sequences of a protease inhibitor found in *Momordica charantia* (Table 1), interestingly all ethanolic protein extracts from *Momordica charantia* seed inhibited trypsin activity higher than purified soybean Kunitz trypsin inhibitor. The 70% ethanol extract containing purified BG-4 peptide inhibited trypsin activity 8.6 times higher than purified soybean trypsin inhibitor. Another interesting observation showed that increasing ethanol concentrations which is equivalent to increasing purity of BG-4 resulted in increased trypsin inhibitory activity (Fig. 1b) indicating that BG-4 is the responsible constituent for trypsin inhibition. Previous reports have shown that trypsin inhibitors from food sources possessed anticancer potential, the most studied are the Bowman-Birk inhibitor and Kunitz trypsin inhibitor from soybean^22^,^23^. Hence we tested BG-4 for its potential anticancer activities using two different human colon cancer cells.

BG-4 dose dependently inhibited the growth of HCT-116 and HT-29 colon cancer cells as shown in Fig. 2a. The cytotoxic effect of BG-4 may be attributed to its capability to inhibit trypsin activity. Previous reports on anticancer potential of trypsin inhibitors showed that mutation on the protease inhibitory unit led to abrogation of the cytotoxic effect of food-derived protease inhibitors. Recombinant Bowman-Birk protease inhibitor isolated from pea dose-dependently inhibited the growth of HT-29 colon cancer cells with an IC_50_ value of 31 μM and the cytotoxic effect was abolished in inactive mutant of the peptide^24^. Previous studies have also shown the anti-proliferative effect of soybean Bowman-Birk inhibitors in HT-29 colon cancer cells indicating an IC_50_ values of 39.9 to 48.3 μM^25^ which is roughly equivalent to 319.2 to 386.4 μg/mL and lentil Bowman-Birk inhibitor in HT-29 colon cancer cells with IC_50_ value of 32 μM equivalent to 240 μg/mL^26^. These values were very similar to our calculated ED_50_ values for HT-29 colon cancer cells of 217.0 to 249.2 μg/mL for BG-4. In addition, administration of *Momordica charantia* in the diet of male Fisher 344 rats led to increased activity of catalase, superoxide mutase and gluthathione-S-transferase with concomitant reduction in the incidence of aberrant crypt foci induced by azoxymethane^27^. These previous studies supported our findings that the anticancer-potential of BG-4 may be attributed to its capability to inhibit trypsin and may be used as either chemotherapeutic or chemopreventative agent against CRC.

We then investigated the mechanism involved in the cytotoxic effect of BG-4 against HCT-116 and HT-29 colon cancer cells. As shown in Figs 4, 5 and 6, the potential mechanistic explanation involved the induction of apoptosis in colon cancer cells. Apoptosis, also known as programmed cell death, is an important machinery for the removal of damaged and mutated cells. Dysregulation in the apoptotic machineries can lead to colon carcinogenesis. In addition, colon cancer cells can develop mechanisms in order to survive and display hallmarks of unlimited cell replication and resistance to cell death^28^. Hence induction of apoptosis in colon cancer cells is an attractive strategy to combat this malignancy. BG-4 treatment at a concentration of 62.5 μg/mL led to increased percentage of apoptotic cells in both HCT-116 and HT-29 colon cancer cells as measured by flow cytometry (Fig. 4). This observation is further supported by fluorescence microscopy (Fig. 5) in which colon cancer cells treated with BG-4 has higher green fluorescence as compared to control untreated colon cancer cells. Apoptosis was activated in human colon cancer cells by modifying the expressions of Bax, Bcl-2, XIAP, caspase-3, p21 and CDK2 proteins (Fig. 6). At 125 μg/mL, BG-4 led to a significant increase in the expression of pro-apoptotic Bax. Bax belongs to the Bcl-2 family of proteins and its activation can lead to mitochondrial outer membrane permeabilization leading to release of pro-apoptotic factors^29^,^30^. The increased expression of Bax may be associated with the capability of BG-4 to reduce the expression of the anti-apoptotic protein Bcl-2. Bcl-2 interacts with Bax, hence reduction in the expression of Bcl-2 causes displacement of Bcl-2 from Bax ultimately leading to Bax activation and apoptosis induction^31^. As a result of reduced Bcl-2 expression and increased Bax expression by BG-4 treatment, the expression of active caspase-3 in human colon cancer cells were significantly increased by 125 μg/mL BG-4 treatment. This increased expression of active caspase-3 may be responsible for the increased percentage of apoptotic cells observed in flow cytometry experiments. Caspase-3 is responsible for executing the apoptotic process and can be inhibited by the anti-apoptotic XIAP^32^. Treatment of BG-4 led to reduced expression of XIAP hence increased expression of caspase-3 in HCT-116 colon cancer cells. In the clinics, expression of pro-apoptotic Bax has been associated with increased survival in advanced CRC patients^33^,^34^ while reduced Bax:Bcl2 ratio was correlated with age and tumor location in CRC patients^35^. Treatment of human colon cancer cells by 125 μg/mL led to increased Bax:Bcl-2 ratio indicating the potential use of BG-4 as anti-colon cancer therapeutic agent. Previous studies on the anti-colon cancer potential of *Momordica charantia* constituents focused on the capability of the isolated fatty acids. For instance, α-eleostearic acid potently inhibited the growth of HT-29 colon cancer cells^36^ and conjugated linolenic acid induced apoptosis by reducing the expression of Bcl-2 with corresponding increase in the expression of GADD45, p53 and PPARγ in Caco-2 colon cancer cells^37^. Another important aspect of cancer cell survival is the capability of cancer cells to multiply as affected by different cell cycle proteins including cyclin dependent kinases and their inhibitors. CDK2 regulates cell cycle transition from G1 to S-phase hence it is important in cell proliferation and was overexpressed in colon cancer cells^38^ while p21 is a CDK inhibitor and a master regulator of tumor suppressor pathways^39^. Our results indicated that BG-4 was able to affect the expression of these proteins important in the survival and proliferation of colon cancer cells further explaining the cytotoxic effect of BG-4 in colon cancer cells *in vitro* by increasing the expression of the antitumor p21 in HT-29 colon cancer cells leading to reduction of CDK2 expression. To our knowledge, this is the first report on the capability of an isolated peptide from *Momordica charantia* to induce apoptosis in colon cancer cells by affecting expression of proteins involved in cell survival and proliferation.

In summary, we first report here a simple method for the isolation and purification of a novel anticancer peptide BG-4 from *Momordica charantia*. In addition, this study reports for the first time the capability of BG-4 to activate apoptosis in human colon cancer cells. Our findings support the idea for the potential use of BG-4 as a colon cancer therapeutic agent and must be studied using *in vivo* models of colon carcinogenesis.

## Methods

### Materials

Bitter gourd (*Momordica charantia* L.) seeds were purchased from Johnny’s Selected Seeds, Maine, USA. Dry seeds of bitter gourd were pulverized to a fine powder under liquid nitrogen with the use of a mortar and pestle and stored at −20 °C until used. All other chemicals were purchased from Sigma-Aldrich (St. Louis, MO) unless otherwise stated.

### Protein isolation

Fifty mg of seed powder was transferred to a 2 mL Eppendorf tubes and extracted with 1 ml of aqueous ethanol on 30 °C shaker for 15 min. The samples were centrifuged at 15,800 × *g* for 10 min and the resulting clear supernatant were saved. To 500 μl of the supernatant 1.5 ml of acetone was added, thoroughly mixed, and placed at −80 °C for 2 h. Precipitated proteins were recovered by centrifugation as above and the resulting pellet was air-dried and resuspended either in 50 mM Tris-HCl buffer, pH 8.2 or in 1X sodium dodecyl sulfate (SDS)-sample buffer (60 mM Tris-HCl, pH 6.8, 2% SDS (w/v), 10% glycerol (v/v), and 5% 2-mercaptoethanol (v/v). Total proteins were isolated from 20 mg of bitter gourd seed powder with 1 ml of SDS sample buffer followed by boiling for 5 min. The clarified supernatant was used for SDS-PAGE analysis.

### Isolation of bitter gourd trypsin inhibitor enriched fraction (BG-4)

Six grams of dry bitter gourd seed powder was extracted with 120 ml of 70% ethanol in an orbital shaker at 30 °C. The slurry was centrifuged at 9,200 rpm for 30 min. To the resulting clear supernatant 3 volumes of ice-cold acetone was added and placed at −20 °C overnight. Precipitated proteins were recovered by centrifugation as above and the pellet was lyophilized. The lyophilized protein pellet was ground to a fine powder with a mortar and pestle and named as BG-4. Protein concentration of the BG-4 was measured using a dye-binding method based on the Bradford assay.

### Sodium Dodecyl Sulfate-Polyacrylamide Gel Electrophoresis

SDS-PAGE analysis was performed as previously described^40^. Bitter gourd proteins were resolved with 15% gels run using a Hoeffer SE 250 Mini-Vertical electrophoresis apparatus (GE Healthcare). Separation was achieved with a constant 20 mA/gel and a typical run time of 1.2 h. Following electrophoresis, the gels were stained with Coomassie Blue R-250.

### Matrix-assisted laser desorption ionization time-of-flight mass spectrometry (MALDI-TOF-MS) analysis

BG-4 kDa protein band was excised from SDS-PAGE gel, thoroughly washed in distilled water, and washed in 50% solution of acetonitrile containing 25 mM ammonium bicarbonate to remove the Coomassie stain. The destained protein band was digested with porcine trypsin and the resulting peptides were analyzed with a LTQ Orbitrap XL hybrid ion trap, Orbitrap mass spectrometer (Thermo Fisher Scientific, San Jose, CA) essentially as described previously^41^. Protein identification was performed using the Mascot search engine (http://www.matrixscience.com) to search against NCBI non-redundant database.

### Trypsin inhibitor assay

Trypsin inhibitory activity was determined as previously described^42^. The assay mixture consists of inhibitor in assay buffer 50 mM Tris–HCl, pH 8.2 containing 20 mM CaCl_2_. Trypsin (10 μg) was added to the assay mixture, mixed, and incubated for 15 min at 37 °C. Following this, 1 mM BAPNA was added to the assay mixture and incubated at 37 °C for additional 10 min. The reaction was terminated by the addition of 30% acetic acid and the absorbance at 410 nm was recorded. One trypsin inhibitory activity unit (TIU) was defined as the amount of inhibitor that reduces the absorbance of the non-inhibited reaction by 0.01.

### Cell culture

HCT-116 and HT-29 (ATCC, Manassas, VA) were kind gifts from Dr. Lee M. Ellis (MD Anderson Cancer Center, University of Texas, Houston TX). These cell lines are not listed on the Database of Cross-contaminated or Misidentified cell lines (ver 7.2 released October 10, 2014 at http://iclac.org/databases/cross-contaminations/) Minimum Essential Medium (MEM) was purchased from Sigma-Aldrich (St. Louis, MO). Heat inactivated fetal bovine serum (FBS) and penicillin-streptomycin solution were purchased from Life Technologies (ThermoFisher Scientific, Waltham, MA). The cells were grown in MEM supplemented with 10% FBS and 1% penicillin-streptomycin in a humidified incubator containing 5% CO_2_ maintained at 37 °C.

### Cell Proliferation Assay

Ten thousand cells were plated in a 96-well plate in 200 μL volume and allowed to attach overnight in humidified 5% CO_2_ incubator at 37 °C. Cells were treated with BG-4 (0 to 1000 μg/mL) for 24 and 48 h. After treatment for indicated time, medium was removed and 20 μL MTS [3-(4,5-dimethylthiazol-2-yl)-5-(3-carboxymethoxyphenyl)-2-(4-sulfophenyl)-2H-tetrazolium, inner salt, Promega, Madison, WI]-PMS (phenazine methosulfate, Acros Organics, New Jersey) solution followed by 100 μL MEM were added. The plate was incubated at 37 °C for 2 h and absorbance read at 490 nm. Proliferation was expressed as percentage of untreated control cells and median effective dose (ED_50_) was calculated using Chou-Talalay method which is based on mass action law principle^43^. All experiments were performed in three independent trials with three replicates per trial.

### Cell morphology

Cells were seeded at 1 × 10^5^ cells/mL in a 6-well plate and allowed to attach overnight in a humidified atmosphere containing 5% CO_2_ at 37 °C. Cells were imaged using phase contrast Invitrogen EVOS FL auto cell imaging system (ThermoFisher Scientific, Waltham MA).

### Colony formation assay

Colon cancer cells were harvested and plated in a 6-well plate at 1,000 cells per well in 2-mL complete growth medium, allow to attach overnight and treated with BG-4 at 62.5 and 125 μg/mL. The cells were incubated for 6 days in a humidified 5% CO_2_ incubator at 37 °C. After 6 days of incubation, medium was removed and colonies were stained with 0.5% crystal violet in methanol for 1 h at room temperature. Crystal violet was removed and plate was placed in a tub of distilled water. After careful washing, plate was left at room temperature in normal air to dry for at least 24 h. Colonies were counted and the effect of BG-4 treatment was expressed relative to the untreated cells. All experiments were performed in two independent trials with two plating per trial.

### Apoptosis Assay

Cells were seeded at 1 × 10^5^ cells/mL in a 6-well plate and allowed to attach overnight in a humidified atmosphere containing 5% CO_2_ at 37 °C. After which, cells were treated with 62.5 and 125 μg/mL BG-4 for 16 h. After treatment, growth medium was removed and cells washed with ice-cold PBS twice and detached from the well. Cell suspension was centrifuged at 500 × g for 5 min at 4 °C and re-suspended in Annexin V binding buffer. Cell suspension (100 μL) was stained with 10 μL Annexin V-FITC (Biotium, Inc., Hayward CA) and 10 μL propidium iodide (Biotium, Inc., Hayward CA) for 15 min in the dark at room temperature. After staining 400 μL of binding buffer was added the suspension was analyzed using BD Accuri C6 flow cytometer (BD Biosciences, San Jose CA). A total of 20,000 events were acquired and analyzed using BD Accuri C6 software package.

### Fluorescence Microscopy

Cells were seeded in an 8-well Millipore EZ-slide chamber dish and allowed to attach overnight. After which, cells were treated with 62.5 and 125 μg/mL BG-4 for 16 h. After treatment, cells were washed twice with binding buffer and stained with Annexin V-FITC for 30 min in the dark at room temperature. After staining and washing with binding buffer, cells were counterstained with Mito-ID^TM^ Red antifade reagent with DAPI (Enzo Life Sciences, Farmingdale NY, USA). Cells were imaged using Invitrogen EVOS FL auto cell imaging system (ThermoFisher Scientific, Waltham MA).

### Western blotting

HCT-116 and HT-29 cells were seeded in 6-well plate at a density of 2 × 10^5^ cells/mL, allowed to attach overnight, treated with BG-4 as mentioned previously and lysed with RIPA buffer. The cell lysates were vortexed for 5 min and centrifuged at 14,000 × g for 10 min at 4 °C. The supernatant was collected, mixed with Laemmli buffer containing 5% β-mercaptoethanol, boiled for 5 min and stored at −20 °C until analysis. A portion of supernatant was diluted with TBS to determine protein concentration as measured using Bicinchoninic acid assay (ThermoFisher Scientific, Waltham MA). The whole cell lysates were used for Bax, Bcl-2, XIAP, caspase-3, p21 and CDK2 (Proteintech Group, Inc. Rosemont IL) and GAPDH (Santa Cruz Biotechnology, Santa Cruz CA) expressions. Western blot analysis was conducted as follows: whole cell lysates (25 μg protein) were loaded in 4–20% Tris-HCl gel (Bio-Rad Laboratories), run at 200 V for 35 min at room temperature. The gels were transferred to PVDF membrane (GE Healthcare Life Sciences, Pittsburgh PA) at 110 V for 1 h at 4 °C. The blots were blocked with 5% non-fat dry milk for 1 h at room temperature, washed three times with TBS containing 0.1% Tween 20 (TBST) for 5 min and incubated with primary antibody overnight at 4 °C at 1:1000 dilution. The blots were washed with TBST five times for 5 min and incubated with anti-rabbit or anti-mouse secondary antibody at 1:5000 dilution for 2 h at room temperature. After washing with TBST, the blots were imaged with C-Digit Blot scanner (Li-Cor Biosciences, Lincoln, NE) using WesternSure ECL Substrate (Li-Cor Biosciences, Lincoln, NE). The intensity of bands was quantified using Image Studio Software (Li-Cor Biosciences, Lincoln, NE). Each experiment was performed for in three independent replicates.

### Statistical Analysis

Data were analyzed using Proc GLM of SAS Software version 9.4 and significance was reported at P < 0.05 with post-hoc Tukey test for separation of means as indicated by different letters. For expression of protein markers related to apoptosis and cell cycle, each concentration of BG-4 treatment was compared against the untreated sample and significance was presented as *(P < 0.05), **(P < 0.01) and ***(P < 0.0001).

## Additional Information

**How to cite this article**: Dia, V. P. and Krishnan, H. B. BG-4, a novel anticancer peptide from bitter gourd (*Momordica charantia*), promotes apoptosis in human colon cancer cells. *Sci. Rep.* **6**, 33532; doi: 10.1038/srep33532 (2016).

## Figures and Tables

**Table 1:** Mass spectrometric identification of amino acid sequences in BG-4 peptide isolated from *Momordica charantia.*

Protein Identified (NCBInr Accession)	MOWSE (50 ppm)	Sequence Coverage	MW (Da)	Peptides Matched (Ion Score)
Glu S. griseus protease inhibitor *Momordica charantia* (gi|114950)	101	50%	7,492	R·SWPQLVGSTGAAAK·A (37)R·VGSPVTADFR·C (49)R·GIVARPPAIG·- (15)
Elastase inhibitor II, MCEI-II serine protease inhibitor *Momordica charantia* (gi|1041917)	92	55%	3,638	R·DSDCLAQCICVDGHCG·-(92)
